# The Role of Diet Diversity and Diet Indices on Allergy Outcomes

**DOI:** 10.3389/fped.2020.00545

**Published:** 2020-09-15

**Authors:** Enza D'Auria, Diego G. Peroni, Marco Ugo Andrea Sartorio, Elvira Verduci, Gian Vincenzo Zuccotti, Carina Venter

**Affiliations:** ^1^Pediatric Department, Vittore Buzzi Children's Hospital, Universitá degli Studi di Milano, Milan, Italy; ^2^Clinical and Experimental Medicine Department, Section of Pediatrics, University of Pisa, Pisa, Italy; ^3^Section of Allergy and Immunology, Children Hospital Colorado, University of Colorado, Denver, CO, United States

**Keywords:** diet diversity, diet indices, pregnancy, allergy outcomes, microbiome

## Abstract

Nutrients in foods are not eaten in isolation and food intake interacts in a complex manner, affecting health and disease outcomes. For this reason, focusing on the whole “pattern” of dietary intake instead of the single nutrients or groups of nutrients when studying diseases outcomes is increasingly appealing and growing. Diet diversity refers to the variety of foods being eaten, and the terms, diversity or variety, are often used interchangeably. When the overall diet is characterized by healthy foods, diet diversity will reflect a diversity/variety of healthy foods eaten over a period of time. The introduction of solid foods in the 1st year of life is considered a measure of increased diet diversity. Consuming a diverse range of foods and food allergens in the first year of life may increase intake of important nutrients and positively affect the gut microbiome structure and function. Intake of omega-3 fatty acids and fibers/prebiotics may be particularly important but more information is required about dose and which individuals are most likely to benefit. Increased diet diversity in the first year of life is also associated with reduced food allergy outcomes. In addition to diet diversity, diet indices are considered measures of overall diet quality and can be used as a simple assessment of dietary intake. The focus of this paper is to review and critically address the current knowledge of the association between diet diversity and diet indices and allergy outcomes. Based on the current evidence, we recommend the introduction of solid foods, including common allergenic solids, during the 1st year of life, according to the infant's neuro-developmental abilities and familial or cultural habits. For infants with severe AD and/or FA, medical assessment may be advisable before introducing common food allergens into the diet. Limited evidence exist about the role of diet indices in pregnancy and allergic disease in the offspring, and the most promising results indicate a reduction in childhood wheeze and/or asthma intake.

## Introduction

Diet diversity is defined as the variety of food being eaten; the term “variety” can be used instead of “diversity” (1). If the diet consists of healthy foods, diet diversity will reflect a diversity/variety of healthy foods eaten over a period of time. Diet diversity may include the number of foods/food groups and the period and the frequency of consumption (2). In this review, we consider the introduction of solid foods in the first year of life as a measure of increased diet diversity. A more diverse diet in the 1st years of life may increase exposure to food allergens, thereby promoting tolerance development (3–7). Diet diversity may also promote an increased intake of nutrients which can be associated with allergic disease prevention. Finally, diet diversity may play a role in allergy prevention by modifying the gut microbiome. During introduction of solid food in the weaning period, higher diet diversity may increase gut microbiome diversity (8). Data regarding the effect of diet diversity in the 1st year of life and atopic dermatitis, rhinitis, and asthma development are conflicting (2).

In this review, we include studies investigating diet diversity, the development of clinical allergy outcomes, and sensitization to aeroallergens and/or foods. In addition to diet diversity, diet indices are considered measures of overall diet quality and offer a simple assessment of dietary intake. In the few last decades, several indices have been developed and employed (9). We focus on diet indices and subsequent development of allergy, with particular emphasis on the Mediterranean diet. We used a combined search strategy using search terms from three EAACI papers (2, 10, 11).

### Aim of the Review

In part 1, we summarize the road map leading to current recommendations on food allergen introduction in the 1st year of life. In part 2, we focus on the effect of diet diversity and diet indices in pregnancy, lactation, and 1st year of life on allergy development.

## Introduction of Solid Foods in the First Year of Life

### History of Introduction of Solid Foods and Food Allergens

Recommendations regarding solid food introduction in early life have changed dramatically in the past two decades. In the early 2000s, the American Academy of Pediatrics (AAP) proposed commencing complementary feeding after first six months and to delay introduction of food allergens until after one year of age in infants considered at high risk for allergy development, such as those having a first-degree relative with a history of allergic diseases. The AAP suggested introduction of milk containing foods after the age 1 year, egg after age two years, and peanuts, tree nuts, and fish after age 3 years (12).

This advice was mainly based on the evidence from two studies; the former (13) showed that early introduction of allergenic foods at 3 months increased risk of atopic disease, and the latter demonstrated a correlation existing between diet diversity before 4 months of life and risk of developing eczema later (14).

In 2006, the American College of Allergy, Asthma, and Immunology (ACAAI) suggested delayed introduction of potential allergenic foods also for children without a risk of atopy/allergic diseases (15). Despite these recommendations, the prevalence of food allergy (FA) continued to increase in Western countries (16). Many observational studies highlighted that postponing the introduction into the diet of foods with an allergenic potential may cause an increased risk of IgE-sensitization and FA (3, 17–22), especially to peanut (3) and egg (19). In support of these studies, the dual allergen hypothesis proposed that early oral food allergen introduction opposed to exposure via the skin might be protective against food allergy (23). This hypothesis is sustained by the fact that skin exposure to food allergens in infants with eczematous skin may favor a Th2 response leading to allergic sensitization (24), whereas oral exposure leads to tolerance.

In 2008, the AAP updated their previous recommendations highlighting that there was not sufficient evidence to postpone introduction into the infants' diet of potential allergenic foods (25). No recommendations were made at that stage about the timing of the introduction of foods. The lack of clear information about food allergen intake at the time was addressed by a number of randomized controlled trials.

### Early Introduction of Allergenic Foods and Food Allergy Prevention

#### Single Allergenic Foods

The Learning Early About Peanut Allergy (LEAP) study by Du Toit et al. (4) demonstrated that introduction of peanut in high-risk atopic infants younger than 1 year old suffering from severe atopic dermatitis and/or egg allergy could reduce the development of peanut allergy. In this study, 640 infants were randomly divided into two groups: some were assigned to consume peanuts, other to avoid peanuts up to 60 months of age. Development of peanut allergy was then tested by an oral food challenge.

The intention to threat analysis showed a significantly lower prevalence of PA in the intervention group than in the control group both in the group with a negative SPT to peanut at the beginning of the study and in children with SPT results of 1–4 mm. Noteworthy, infants (7/640) who had never been fed peanuts previously had positive oral peanut challenge at enrolment both in the case of positive SPTs (6 out of 47, 12.8%) and negative SPTs (1 out of 272, 0.4%).

The authors used the term “early introduction” reflecting introduction between 4 and 11 months, differently from the delayed introduction (after 2 years of age), previously recommended in international guidelines (12), indicating the importance of introducing peanut in the 1st year of life and continuing with regular peanut intake once introduced.

The LEAP-On follow-up study showed that cessation of peanut intake for 1 year, after consumption for 5 years, did not lead to a significant increase of peanut allergy by 6 years of age (26).

The ongoing Preventing Peanut Allergy in Atopic Dermatitis (PEAAD) trial is evaluating if peanut ingestion for one year, in infants and children aged 5–30 months and suffering from AD, may have an effect on the development of peanut allergy (27).

Several randomized controlled trials (RCTs) focused on clarifying whether the earlier introduction (before 6 months of age) of other potential allergenic foods (e.g., egg) into diet may prevent the occurrence of developing allergic sensitization and FA (4, 26, 28–32). These RCTs are summarized in Table 1.

**Table 1:** Food allergy prevention via early introduction of allergenic food: list of RCTs.

**Trial, Year, Country**	**Study type**	**Food type**	**Inclusion criteria**	**Type of intervention (no. of subjects)**	**Outcomes**	**Results**
STAR (30), 2013, Australia	DBPCRCT	Egg (raw white pasteurized)	4 m.o. infants at risk for allergy (moderate-severe eczema—SCORAD ≥15)	Intervention (33): 0.9 g of egg protein per day from 4 to 8 months Control (34): placebo	Primary: prevalence of OFC-diagnosed EA at 1 year of age	A lower but not significant proportion of infants in the egg group (33%) had EA compared with the control group (51%; relative risk, 0.65; 95% CI, 0.38–1.11; *P* = 0.11)
EAT (32), 2014, UK	RCT	Milk (yogurt), cooked egg, wheat, peanut, sesame, fish	3 m.o. infants exclusively breastfed, not at risk for allergy	Intervention (652): from 3 to 6 months sequential introduction of the six foods (milk always first) 4 g proteins/week Control (651): only breastfed from birth to 6 months of age	Primary: prevalence of OFC-diagnosed FA to one of the six foods at 1–3 years of age	In the intention-to-treat analysis, no statistically significant difference between early introduction group and standard introduction group
LEAP (4), 2015, UK	RCT	Peanut (snack/butter)	4–11 m.o. infants at risk for allergy (moderate-severe eczema and/or EA, SPT ≤ 4 mm)	Intervention (319): 6 g peanut protein/week, ≥ 3 times/week, up to 5 years of age Control (321): peanut avoidance	Primary: prevalence OFC-diagnosed PA at 5 years of age	Among SPT-negative infants, prevalence of PA at 60 months of age was 13.7% in the avoidance group and 1.9% in the consumption group (*P* < 0.001) Among SPT-positive infants, prevalence of PA was 35.3% in the avoidance group and 10.6% in the consumption group (*P* = 0.004)
HEAP (28), 2017, Germany	DBPCRCT	Egg (raw white pasteurized)	4–6 m.o. infants not at risk for allergy (egg s-IgE <0.35 kU/L)	Intervention (184): 2.5 g of egg protein at least 3 times per week starting from 4 to 6 months until 12 months of age Control (199): placebo	Primary: egg s-IgE positivity Secondary: prevalence of OFC-diagnosed EA at 1 year of age	5.6% of the children in the egg group were hen's egg sensitized and 2.1% were confirmed to have hen's EA vs. 2.6 and 0.6%, respectively, in the placebo group (For sensitization: RR, 2.20; 95% CI, 0.68–7.14; *P* = 0.24) (For allergy: RR, 3.30; 95% CI, 0.35–31.32; *P* = 0.35)
STEP (31), 2017, Australia	DBPCRCT	Egg (raw whole pasteurized)	4–6 m.o. infants at risk for allergy (maternal atopy history, no eczema), who have never taken egg	Intervention (407): 0.4 g of egg protein per day starting from 4 to 6 months until 10 months of age Control (413): placebo From 10 months, no restriction for egg introduction in both intervention and placebo groups	Primary: prevalence of OFC-diagnosed EA at 1 year of age	No difference in term of IgE-mediated EA (egg 7.0% vs. control 10.3%; adjusted relative risk, 0.75; 95% CI, 0.48–1.17; *P* = 0.20)
BEAT (35), 2017, Australia	DBPCRCT	Egg (raw whole pasteurized)	4 m.o. infants at risk for allergy (≥1 first degree relative with allergy)	Intervention (165): 0.35 g of egg protein per day starting from 4 to 8 months of age Control (154): placebo From 8 months, no restriction for egg introduction in both intervention and placebo groups	Primary: egg white SPT positivity Secondary: prevalence of OFC-diagnosed EA at 1 year of age.	Sensitization to EW at 12 months was 20 and 11% in infants randomized to placebo and egg, respectively (odds ratio, 0.46; 95% CI, 0.22–0.95; *P* = 0.03, χ^2^ test)
PETIT (36), 2017, Japan	DBPCRCT	Egg (cooked lyophilized)	4–5 m.o. infants at risk (eczema), who have never taken egg	Intervention (37): 0.025 g of egg protein per day between 6 and 9 months, followed by 0.12 g between 9 and 12 months of age Control (38): placebo	Primary: prevalence of OFC-diagnosed EA at 1 year of age	8% of egg group had an EA compared to 38% of placebo group (RR 0.221 [0.090–0.543]; *p* = 0.0001)

*DBPC, Double-blind, placebo-controlled; EA, Egg allergy; EW, Egg White; OFC, Oral Food Challenge; PA, Peanut allergy; RCT, Randomized Clinical Trial; RR, Relative risk*.

Studies evaluating the effects of early introduction of egg gave contrasting results, probably related to variation in the study design (populations, outcomes) and in the dose and the form of egg used (i.e., cooked vs. raw egg; Table 1).

The Solid Timing for Allergy Research (STAR Study) (30) trial reported that early introduction of egg had no protective effect on the development of EA in high risk infants who had never eaten egg. The study was discontinued because of a high rate of allergic reactions to pasteurized raw egg. Since then, two RCTs showed no difference in the development of egg sensitization or EA in high risk infants consuming or avoiding egg.

The Australian Study Starting Time of Egg Protein (STEP) trial (31) considered 820 at risk infants (e.g., infants with atopic mothers) who had never consumed egg and did not show allergic symptoms. Infants were randomly allocated to consume pasteurized raw egg or placebo from 4 to 6 months of life until 10 months. No differences were found at the intention-to-treat (ITT) analysis between the active and placebo groups in terms of OFC-diagnosed EA development (7% active vs. 10.3% control) and in cutaneous sensitization, defined by positive egg SPT (10.8% active vs. 15.1% control, *P* = 0.15) at 12 months.

The Beat Egg Allergy Trial (BEAT) (35) randomized children at high risk (having at least a first-degree relative with atopic disease), not sensitized, to receive pasteurized whole raw egg powder or placebo from 4 to 8 months of age. At 12 months, no difference was observed for the percentages of positive challenge between the two groups (10.5% active vs. 6.2% placebo), despite a lower prevalence of sensitization to egg white in the active group.

In the Hen's Egg Allergy Prevention (HEAP) (28) infants aged 4–6 months without risk factors for allergy development were recruited and randomly assigned to be fed pasteurized raw egg white powder or placebo. At 12 months of age, no difference was observed in the prevalence of EA or egg specific-IgE between the two groups.

The most recent Prevention of Egg allergy with Tiny Amount Intake (PETIT) (36) trial assessed the safety and efficacy of a step-wise introduction of heated egg (equivalent to 0.2 g of whole egg boiled for 15 min) in a sample of infants with mild to severe atopic dermatitis, no immediate allergic reaction to egg, and no history of immediate allergic reaction to egg or to any type of food. All infants were treated with topical treatment for AD during the study.

The results of the study showed a statistically significant decrease of OFC-diagnosed EA at 12 months of age in the study group (8% intervention vs. 38% control, *p* < 0.0001).

However, it should be pointed out that only per protocol analysis was conducted and the primary outcome (OFC-diagnosed EA) was not established in 26 (17%) infants; thus, these findings need to be treated with caution.

A recent meta-analysis of RCTs (39) (Table 1) showed with moderate evidence that egg introduction at the age of 4–6 months reduces EA occurrence. However, the conclusions of this meta-analysis mainly relied on the results of the PETIT study (36), in which the stepwise introduction of cooked egg from six months of age seemed to be effective in reducing EA prevalence.

A recent RCT showed that avoiding cow's milk formula supplementation in newborns for the first 3 days of life reduce sensitization to cow's milk and food allergy (40).

Similarly, a Finnish cohort study (*n* = 6,200 infants), with a follow-up of 18–34 months, found that exposure to cow's milk (CM) proteins within the first few days of life increased the risk for CM allergy (34). In contrast, a cohort study from Israel reported that the introduction of CM proteins within the first 2 weeks of life was associated to a lower risk of CM allergy development, and introduction between 4 and 6 months of life was associated to a higher risk (40).

Omega-3 fatty acids (present in fatty fish) have anti-inflammatory properties. Observational studies investigated if a relationship exists between the timing of fish introduction and the risk of subsequent asthma and atopic diseases (41–45). Pooling the results of these studies showed that there is limited evidence to support early fish introduction (before 9 months of life) for reducing the risk of allergic sensitization, rhinitis (35), and asthma (46). Nevertheless, fish contains important nutrients and omega-3 fatty acids play an important role in development of the central nervous system (47, 48). We feel reasonable to recommend fish introduction during the second semester of life, timing based on local tradition, weaning approach, and family preferences (49, 50).

In summary, the introduction of peanuts from 4 to 11 months of age in infants at high risk of developing PA may be beneficial to prevent peanut allergy. The same may be true for introduction of heated egg. With regard to early introduction of other allergenic foods (milk, fish, and cereals) most of the available data are from observational studies and do not prove a cause-effect relationship.

#### Multiple Allergenic Foods

The Enquiring About Tolerance (EAT) is the only intervention trial which aimed to investigate the effects on allergy development of early ingestion of different food allergens (i.e., milk, peanut, egg, wheat, fish, and sesame) in exclusively breastfed infants with unknown risk of allergy status (32).

The per protocol analysis revealed a statistically significant decrease of overall FA (2.4 vs. 7.3%, *p* = 0.01), PA (0 vs. 2.5%, *p* = 0.003; NNT 40) in the early introduction group compared to the control; the ITT analysis did not show a different occurrence of FA to at least one of the six foods at three years follow-up. In the per protocol analysis, the NNT was very high. The findings of this study suggested a possible effect on FA prevention with introduction of foods and food allergens between 3 and 6 months of age. However, non-adherence rate in the intervention group was substantial (68.1%) and may lead to bias in the per-protocol analysis (51). It also indicated the difficulty for parents to introduce so many allergens at such an early age (33). Of note, the lowest adherence rate was reported for the introduction of egg (43.1%).

In many of these studies, infants were sensitized just before introducing the allergenic food/s (4, 24, 26, 27). This suggests that other factors, including genetics, epigenetics, and gut flora, could take part in FA development before weaning (52–56).

### Current Recommendations

In response to the Learning Early About Peanut (LEAP) study (4) and a number of RCTs on egg and multiple allergen introductions, guidelines around the world were adapted. The first were the ASCIA (57) guidelines suggesting that peanut, cooked egg, wheat, and dairy foods should be introduced into infants' diet in the 1st year of life. The NIAID (USA) guidelines (2017) (58) suggested different peanut introduction schedules depending on the degree of risk: in infants affected by severe atopic dermatitis and/or EA from 4 to 6 months while around 6 months in infants with mild to moderate eczema and that family and cultural feeding practices should be followed in infants with no eczema or any food allergies. The latest COT report (UK) (59) and BSACI guidance (60) suggest to introduce complementary foods, in the general population, from around 6 months of age, including peanut and egg. The BSACI guidance suggests that in *high risk* infants, parents may wish to start complementary feeding around 4 months of age. Parents may also consider to include egg and peanut, but current guidelines are in disagreement about the feasibility and need for assessment prior to introduction.

The most recent update AAP guidelines (61) state that there is no evidence for postponing food allergen introduction beyond 4–6 months, including allergenic foods.

In summary: Evidence suggests introduction of peanut and egg before 11 months of age in high risk infants, after medical assessment. These foods can be given also as part of the weaning diet in low risk infants, ideally before 1 year. There is no/limited evidence regarding the other food allergens but it does not suggest to delay introduction of these foods unnecessarily (Table 2).

**Table 2:** Timing of the introduction of potential food allergens.

**Medical societies and scientific societies**	**Year**	**Recommendation**
AAP (25)	2008	No evidence that delaying introduction of solid food beyond 4–6 months of age has a significant protective effect on the development of atopic disease regardless of whether infants are fed cow milk protein formula or human milk.
AAAAI (62)	2013	Complementary foods can be introduced between 4 and 6 months of age.
ASCIA (57)	2016	Complementary food to be introduced at around 6 months, but not before 4 months
ESPGHAN (63)	2017	Complementary foods (i.e., solid foods and liquids other than breast milk or infant formula) should not be introduced before 4 months but should not be delayed beyond 6 months. Allergenic foods may be introduced when complementary foods is commenced any time after 4 months.
NIAID (58)	2017	For peanut Severe eczema and/or egg allergy: between 4–6 months Mild to moderate eczema: around 6 months in infants No eczema or any food allergies: according to family and cultural feeding practices
BSACI/BDA (64)	2018	No risk factors for food allergy: introduce solid foods at around 6 months of age but not before 4 months, including peanut, egg, and other foods eaten as part of the family's normal diet. Eczema or existing food allergy: consider introducing solid foods including cooked egg and then peanut from age 4 months, alongside other solids
AAP (61)	2019	There is no evidence that delaying the introduction of allergenic foods, including peanuts, eggs, and fish, beyond 4 to 6 months prevents atopic disease.

*AAP, American Academy of Pediatrics; AAAAI, American Academy of Allergy, Asthma and Immunology; ASCIA, Australasian Society of Clinical Immunology and Allergy; BDA, British Dietetic Association; BSACI, British Society for Allergy and Clinical Immunology; ESPGHAN, European Society for Pediatric Gastroenterology, Hepatology, and Nutrition; NIAID, National Institute of Allergy and Infectious Diseases*.

## Diet Diversity in Pregnancy

The role of diet diversity in pregnancy and offspring outcomes has not been studied.

## Diet Diversity in the 1st Year of Life and Allergy Outcomes

The PASTEUR/EFRAIM Study by Carole Roduit et al. (37) is the first study specifically describing the role of diet variety in early life and its effect on food allergy development. This prospective, multicenter study evaluated the association between complementary food introduction in the 1st year of life and allergy sensitization or clinical outcomes up to 6 years of age. In this study, diet variety was defined by investigators in two ways: Definition 1: group of 15 different foods frequently assumed by 80% of the study population in the first 12 months of age. Definition 2: group of the 6 major foods introduced in the first 6 months or first 12 months of life. The risk of sensitization to food allergens at the age of 4.5 or 6 years was higher in the group of children with lower diet diversity. The same study also showed a reduction in reported doctor-diagnosed food allergy up to 6 years of age associated with increased introduction of vegetables/fruits, cereals, bread, meat, cake, and yogurt within the first 6 months or first 12 months of life.

In addition to this study, Venter et al. (38) recently reported an association between increased diet diversity in the first year of life and reduced odds of food allergy over the first decade of life. In particular, they showed that the introduction of each additional food at 6 months of age reduced by 10.8% the odds of developing food allergy over the first 10 years of life. Moreover, for each additional food allergen introduced by 12 months, it reported a significant reduction of 33.2% in the likelihood of food allergy over the first 10 years of life. These studies may indicate that diet diversity is associated with increased nutrient intake, including those nutrients which could have a protective role on allergy development (omega-3 fatty acids and non-digestible fibers) (65, 66). Other studies investigated the effect of diet diversity in early life and allergy outcomes are summarized in Table 3.

**Table 3:** Allergy prevention via diet diversity increase: list of studies.

**Study/Author, Year, Country**	**Study type**	**Sample size**	**Food diversity assessment**	**Outcomes**	**Results**
PASTURE (37), 2014, Austria, Finland, France, Germany, Switzerland	Prospective birth cohort	Baseline: 1,133 Analytic: 856	Parent self-administered questionnaire on dietary intake at 2, 12, 18, and 24 months of age and then yearly up to age 6 years	Food allergy (parental report of ever doctor-diagnosed food allergy up to 6 years of life)	Increased DD in the 1st year of life associated with reduced risk of reported doctor-diagnosed food allergy up to 6 years
				Asthma (at least one either doctor-diagnosed asthma or at least 2 doctor-diagnosed episodes of obstructive bronchitis in the last 12 months in the year 4, 5, or 6 questionnaires independent of a diagnosis reported in the first 3 years of life)	Increased DD in first year of life associated with reduction of reported asthma (for each successive food introduced: 26% reduction)
				Allergic rhinitis (reported presence of symptoms or doctor-diagnosed allergic rhinitis in the 6-year questionnaire)	No effects of DD on allergic rhinitis development
				Atopic dermatitis (parental report of doctor-diagnosis up to 4 years and/or positive SCORAD score during medical examination at the age of 1 year)	Increased DD within first year of life associated with reduction of AD development up to 4 years of age (for each successive food introduced in 1st year: 25% reduction)
Finnish Type I Diabetes Prediction and Prevention Study (67), 2014, Finland	Prospective Cohort Study	Baseline: 4,074 Analytic: 3,781	Age-specific dietary questionnaires at ages 3, 4, 6, and 12 months	Atopic dermatitis (parental reports of doctor-diagnosis ever up to 5 years)	No effects of DD at 3 and 4 months of age and AD, development up to 5 years. DD in 1st year of life positively associated with reduction in asthma up to 5 years. DD in first year of life not associated with reduction in AR up to 5 years. Reduced DD at 6 months associated with increased risk of AD development up to 5 years
LISA Study, 2006-2008 (21, 22), Germany	Prospective birth cohort study	2612 up to 2 years, 2073 up to 6 years	Parental interview on infant's diet at 6 and 12 months	Atopic dermatitis (parental reports on doctor-diagnosis and symptoms of AD, questionnaires at birth, 0.5, 1, 1.5, 2, 4, and 6 years)	Increased DD in the first 6 months associated with reduced risk of AD development at 2 years No effects of DD at 4 months and AD risk Excluded infants/children with early symptoms of allergy, increased DD associated with an increased risk of doctor-diagnosis of AD, but no symptoms of AD at 6 years
GINI Study (17), 2007, Germany	Prospective Birth Cohort Study	4,753	Parental interview on infant's diet at 12 months	Parental reports on doctor—diagnosis and symptoms of AD, yearly up to 4 year	No significant association between AD (both doctor-diagnosed or symptomatic) and diversity of solids
GINIplus and LISAplus study (68), 2011, Germany	Prospective Birth Cohort Study	9,088	Parental report at 4 and 6 months	See LISA and GINA study	No association between DD at the age of 4 and 6 months and AD (doctor-diagnosed) in the first 4 y of life High DD before 17 weeks of age may be associated with an increased allergy development Increased DD at 4 months associated with higher risk of symptomatic AD at 2 years, doctor-diagnosed AD at 6 years, but not at 4 years
LISAplus (69), 2017, Germany	Prospective Birth Cohort Study	Baseline: 3,097 Analytic: 2,518	Self-administered parental questionnaires from birth to 2 years of age and at 4 and 6 years	Doctor-diagnosed eczema, asthma, and allergic rhinitis assessed at 1 year of age	Children in the highest quartile who were introduced to all eight food groups during the 1st year of life had lower odds of developing eczema up to age 15 years compared with children in the lowest quartile with a maximum of five food groups
Turati (70), 2017, Italy	Case-control study	451 (+451 controls)	Face to face questionnaire for number of items introduced in the infant's diet at 4 and 5 months	Dermatologist-diagnosed AD between 3 and 24 months	The introduction of a high number of different solid foods at 4 and 5 months of age reduced the risk of AD
Fergusson, 1981-1994 (14, 71–73), New Zeland	Prospective Birth Cohort Study	1,265–1,067	Parent interview and food diary assessed at 4 months	Maternal report, doctor report	Increased DD at 4 months was associated with increased eczema at 2 years, increased risk for AD at 2 years, increased risk for AD up to 3 years, increased risk of recurrent/chronic AD up to 10 years

### Omega-3 Fatty Acids

Omega-3 long-chain polyunsaturated fatty acids (LCPUFAs) are essential nutrients found in many foods such as fatty/oily fish, fish oil, and nuts (66, 74, 75). Evidence from *in vitro* and *in vivo* studies have shown that omega-3 fatty acids are able to lower pro-inflammatory cytokines and antagonize IgE responses and mast cells degranulation (66, 74). An imbalance in omega-6 fatty acid intake vs. omega-3 fatty acid in people eating a Western Diet (76, 77) has been considered as a possible reason for the increase in allergic diseases. This may be due to the pro-inflammatory activity of omega-6 fatty which favors a Th2 immune response (78).

However, other than some effect on allergen sensitization, evidence from RCTS is not conclusive to support omega-3 fatty acid supplementation for offspring allergy prevention during pregnancy and/or lactation (65, 66). Omega-3 fatty acid supplementation has also been studied in infancy and childhood with conflicting results. Using house dust mite (HDM) avoidance and dietary fatty acid modification during the first 5 years of life, the Childhood Asthma Prevention Study (CAPS) showed modification of the plasma fatty acids status (increasing omega-3/omega-6 ratio) at age 5 years, but no clinical effects (79).

D'Vaz et al. (80) showed that in infants at high risk of atopy, supplementation of omega 3 fatty acids for the first 6 months of life had beneficial effects on preventing sensitization, eczema, and food allergy. Similarly, Birch et al. (81) demonstrated that infant formula supplemented with omega-3 fatty acids in healthy infants is protective against allergic disease (wheezing/asthma, wheezing/asthma/atopic dermatitis, or any allergy) throughout three years of life. The difference in findings of these studies may be explained by differences in the underlying diet, high risk/low risk populations, dose of supplements used, timing and duration of supplementation, or serum levels of omega-3 intake at the start of the trial (75, 79, 80, 82, 83).

### Non Digestible Fibers/Prebiotics

Prebiotics are defined as a “substrate that is selectively utilized by host microorganisms conferring a health benefit” (84). They naturally occur in foods or can be artificially produced as galactooligosaccharides (GOS) and fructooligosaccharides (FOS). In the large bowel, prebiotics undergo fermentation by local bacteria modulating the microbiota composition. Microbiota can, in turn, produce beneficial metabolites such as short-chain fatty acids with known anti-inflammatory effects. Human milk contains more than different 400 oligosaccharides which act also as prebiotics (66) and can shape infant gut microbiota composition (85). Following from this, prebiotics have been added to infant formula trying to mimic the beneficial effect of breastmilk (85).

A recent metaanalysis including 22 studies assessed the effect of supplementing prebiotics in infants on the risk of development of allergic symptoms. Studies on infants at high risk and normal risk of allergy were included. Most of these studies evaluated FOS with GOS supplementation added to infant formula. The authors concluded that the evidence for supplementation of prebiotics for the prevention of allergies are not strong enough to make any clear recommendations (86).

Focusing on the whole “pattern” of dietary intake instead of the single nutrients or groups of nutrients when studying diseases outcomes is therefore increasingly appealing and growing. This is because nutrients and foods are not eaten in isolation. All food intake interacts in a complex manner to determine well-being or disease.

## Diet Diversity and the Microbiome

If there is a place for diet diversity in allergy prevention, then the mechanisms of action need to be clarified. Diet diversity may affect the gut microbiome by providing a more diverse food intake which may increase intake of fiber and other nutrients affecting the gut microbiome. Very few studies have compared diet diversity to gut microbiome diversity. The first study conducted by Claesson et al. (87) showed that increased diet diversity in the elderly was associated with increased gut microbial diversity. If introduction of solid food is considered an increase in diet diversity, then this would be another example of how diet diversity increases gut microbiome diversity. This was reflected by increased protein intake, carbohydrate, and fiber intake, as well as in increased intake of family foods (88). Increased gut microbial diversity has been related to reduced allergen sensitization (89) and allergy outcomes in both children and adults (90–92).

## Diet Indices in Pregnancy

Three diet indices in pregnancy have been studied in relation to allergy outcomes in infants, the healthy eating index (93–95), the diet inflammatory index (95, 96), and the Mediterranean diet index (93, 97–100) (Table 4).

**Table 4:** Diet indices in pregnancy and allergy outcomes in the offspring.

**References**	**Exposure**	**Outcomes**
Castro-Rodriguez et al. (97)	Mediterranean diet score Spain *N* = 1,000 Age assessed: 1.5 and 4 years	Current wheezing, rhinitis, and dermatitis MedDiet scores during pregnancy was not a protective factor for current wheezing, rhinitis, or dermatitis in preschoolers.
Chatzi et al. (98)	EPIC scores used with the addition of milk and removal of alcohol Spain, Greece *N* = 1,771 Age assessed: 1 year	Wheeze and Eczema EPIc scores was not associated with the risk of wheeze and eczema in any cohort.
Chatzi et al. (99)	EPIC scores used with the addition of milk and removal of alcohol Spain *N* = 460 Age assessed: 6.5 years	Persistent wheeze, atopic wheeze, atopy (sensitization to ≥1/6 common aeroallergens) Higher EPIC scores were negatively associated with persistent wheeze OR 0.23 (0.09–0.60), atopic wheeze OR 0.34 (0.12–0.97) and atopy OR 0.55 (0.32–1.97)
Bedard et al. (100)	Mediterranean diet score United Kingdom Avon Longitudinal Study of Parents and Children (ALSPAC) *N* = 8,907 Age assessed = 7.5 years	Asthma, eczema, maximal mid-expiratory flow The maternal Mediterranean diet score was not associated with asthma or other allergic outcomes Weak positive associations were found between maternal Mediterranean diet score and childhood maximal mid-expiratory flow after controlling for confounders Higher Mediterranean diet scores were associated with increased FEF_25−75%_ *z*-scores adjusted for age, height, and sex (β 0.06, 95% CI 0.01–0.12; *p* = 0.03, comparing a score of 4-7 vs. a score of 0–3)
Lange et al. (93)	Mediterranean diet score and Alternate Healthy Eating Index modified for pregnancy (AHEI-P) USA *N* = 1376 Age assessed: 3 years	Recurrent wheeze None of these indices, recurrent wheeze in children Secondary outcomes: Doctor's diagnosis of asthma, eczema, lower respiratory tract infections, or atopy at any time (0–3 years); in the adjusted models, neither diet score was associated with any of the secondary outcomes
Moonesinghe et al. (94)	Alternate Healthy Eating Index modified for pregnancy (AHEI-P) UK *N* = 937 Age assessed: 3 and 10 years	Atopy (sensitization to any food and/or aero-allergen), reported allergic diseases (asthma, eczema, allergic rhinitis, and food allergy) AHEI-P was not associated with atopy or allergic diseases
Chen et al. (95)	Diet inflammatory index and Healthy Eating index (HEI-2015) Ireland *N* = 862 Age assessed: first 10 years of life	Asthma for the first 10 years of life: Higher diet inflammatory diet scores were associated with higher risk of offspring asthma (OR: 1.35; 95% CI: 1.10, 1.65). Higher HEI-2015 scores were associated with lower risk of asthma (OR: 0.77; 95% CI: 0.64, 0.93) (both *P* < 0.01); persisted in adjusted models
Hanson et al. (96)	Diet inflammatory index USA *N* = 1,424 Age assessed: first 9 years of life	Ever asthma and wheezing in the past year (early childhood and mid childhood); current asthma and lung function (mid childhood), and wheeze trajectory during 1–9 years Higher diet inflammatory scores were associated with an early vs. never wheeze trajectory (OR, 1.89; 95% CI, 1.14–3.13) (adjusted models) and lower forced expiratory flow (forced expiratory flow at 25-75%) in mid childhood (beta, −132 mL; 95% CI, −249 to −14). Ever asthma, were not related to diet inflammatory scores

### The Healthy Eating Index

Three studies have studied the role of healthy eating in pregnancy and childhood allergy outcomes. In one study (Food Allergy and Research Study, UK), the Alternate Healthy Eating Index modified for pregnancy (AHEI-P) was used to examine associations with allergic outcomes in the offspring at 3 and 10 years (94). This study found no association between the AHEI-P and atopy (defined as sensitization to any food and/or aero-allergen) or reported allergic diseases at 3 or 10 years. Lange et al. (93) used data from the Project Viva cohort (US) and found no association between the HEI and recurrent wheeze in infants at the age of 3 years. In contract, Chen et al. (95) using data from an Irish cohort found that higher HEI-2015 scores were associated with lower risk of asthma (OR: 0.77; 95% CI: 0.64, 0.93) (both *P* < 0.01) and the effect persisted in adjusted models.

### The Diet Inflammatory Index

Two studies have looked at the association between a pro-inflammatory diet in pregnancy and asthma, wheeze, or lung function outcomes through childhood (95, 96). One recent study from Ireland (95) showed an association between DII scores and asthma outcomes over 10 years. The Project viva (US) study showed an association of DII scores with wheeze trajectories, but not asthma, up to 7.5 years of age (96).

### The Mediterranean Diet Index

The associations between the Mediterranean diet index and allergy outcomes have been studied in by five cohorts (93, 97–99) (two from Spain, one from Greece, one from the USA, and one from the UK). Four studies investigated wheeze in the infant (93, 97–99), four studies investigated rhinitis, atopic dermatitis, and/or eczema (97–100), two studies included sensitization to food/aero-allergens (93, 99), and two studies investigated asthma in the child (93). Four of the five studies found no association between the Mediterranean diet index and the allergic outcomes studied (93, 97, 98, 100). Childhood persistent wheeze, atopic wheeze, and atopy was associated with the Mediterranean diet index in only one of the studies (99). One study (100) found an association between the Mediterranean diet score and childhood maximal mid-expiratory flow as well as FEF_25−75%_ *z*-scores.

Based on the current evidence, limited recommendations can be made about the role of diet indices in pregnancy and allergy outcomes in the offspring. The most potential of these indices may be in the prevention of lung function, wheeze, or asthma outcomes in the offspring.

## Diet Indices in Childhood

The role of diet indices in infancy vs. subsequent development of allergic outcomes in later childhood have not been conducted. A number of studies have however looked at the Mediterranean diet in childhood vs. allergy outcomes, using either the KIDMED mediterranean score (99, 101–106) or the adult EPIC score (107–115) and the diet inflammatory index (116, 117) (Table 5).

**Table 5:** Diet indices in childhood and allergy outcomes.

**References**	**Exposure**	**Outcomes**
**KIDMED—Mediterranean Diet Quality Index for children and teenagers**		
Grigoropoulou et al. (106)	Greece *N* = 1,125 Age assessed: 10–12 years	Asthma 1-unit increase in the Kidmed index was associated with 16% lower likelihood of having asthma symptoms Greater adherence to MD was inversely associated with “ever wheeze” (O: 0.88; 95% CI, 0.78, 0.98) and wheeze when exercise w (OR: 0.79; 95% CI, 0.67, 0.93).
Chatzi et al. (99)	Spain *N* = 460 Age assessed: 6.5 years	Persistent wheeze, atopic wheeze (current wheeze and atopy), atopy (sensitization to ≥1/6 common aeroallergens) No statistical significant effect was seen.
Alphantonogeorgos et al. (101)	Greece Urban (Athens, *n* = 700) or rural environment (*n* = 425) Age assessed: 10–12 years	Asthma, any asthmatic symptom Adherence to the Kidmed index was negatively associated with asthma symptoms (standardized beta = −0.224, *p* < 0.001).
Arvaniti et al. (102)	Greece *N* = 700 Age assessed: 10–12 years	Ever asthma, any asthma symptoms, ever wheeze, exercise induced wheeze, night cough Greater adherence to MD inversely associated with ever asthma (*p* = 0.002), any asthma symptoms (*P* < 0.001), ever wheeze (*p* < 0.001), exercise induced wheeze (*p* = 0.004). One-unit increase in KidMed score was associated with 14% lower likelihood of having asthma.
Chatzi et al. (104)	Greece *N* = 690 Age assessed: 7–18 years	Respiratory and allergic symptoms over the past 12 months, skin prick tests to 10 aeroallergens, any wheezing in the past, atopic wheeze, current wheezing, nocturnal dry cough, any rhinitis in the past, atopic rhinitis, current allergic rhinitis, current seasonal rhinitis, atopy High level of adherence to MD was inversely related to Allergic rhintis ever OR 0.34 (0.18–0.64) *p* < 0.01 Allergic rhinitis with atopy OR 0.39 (0.13–0.97) Current allergic *h* = rhinitis OR 0.49 (0.24–0.99) *p* < 0.05 Nocturnal cough apart from cold in the last 12 months OR 0.49 (0.23–0.96) No significant effect seen for wheezing and atopy
**EPIC—European Prospective Investigation into Cancer and Nutrition Cohort—Mediterranean diet score**		
Castro-Rodriguez et al. (107)	Spain *N* = 1,784 Age assessed: 08 ± 0.8 years	Current wheezing Highest quartile of EPIC scores associated with a reduction in current wheeze 0.54 (0.33–0.88)
de Batlle et al. (108)	Mexico *N* = 1,476 Age assessed: 6–7 years	Asthma ever, wheeze ever, current wheeze, rhinitis ever, sneezing ever, current sneezing, current itchy-watery eyes. rhinitis related outcomes by ISAAC questionnaire Adherence to the EPIC scores (2nd and 3rd tertile compared with 1st tertile) inversely associated with asthma ever OR 0.60 (0.40–0.91), wheezing ever OR 0.64 (0.47–0.87), current Sneezing OR 0.71 (0.52–0.96) and current itchy-watery eyes OR 0.63 (0.42–0.95)
Garcia-Marcos L et al. (109)	Spain *N* = 20,106 Age assessed: 6–7 years	Current occasional asthma, current severe asthma, rhinoconjunctivitis Every 1 unit increase in EPIC score showed a protective effect on current severe asthma in girls (adjusted OR 0.90, 95% CI: 0.82–0.98)
Suarez-Varela et al. (110)	Spain *N* = 20,106 Age assessed: 6–7 years	Atopic dermatitis No association between EPIC diet scores and atopic dermatitis
Tamay et al. (111)	Turkey *N* = 9,875 Age assessed: 6–7 years	Allergic rhinitis, lifetime rhinitis, current rhinitis, current rhinoconjunctivitis, physician-diagnosed allergic rhinitis No association between EPIC diet scores and any of the outcomes studied
Akcay et al. (112)	Turkey *N* = 9,991 Age assessed: 13–14 years	Wheeze ever, wheezing in last 12 months, lifetime doctor diagnosed asthma prevalence No association between EPIC diet scores and any of the outcomes studied
Rice et al. (113)	Peru *N* = 287 asthmatic + 96 controls Age assessed: 9–19 years	Asthma status (asthma control, FEV1), allergic rhinitis, atopy No association between EPIC scores and asthma control, FEV1, allergic rhinitis, or atopic status
Romieu et al. (114)	Mexico *N* = 158 asthmatic + 50 controls Age assessed: 6–14 years	Pulmonary function was measured and nasal lavage collected and analyzed. No significant difference between the asthmatic and the non-asthmatic children.
Gonzalez et al. (115)	Spain *N* = 7,454 Age assessed: 6–7 years *N* = 7,391 Age assessed: 13–14 years	6–7 years: Asthma current asthma, severe asthma, and exercise-induced asthma 13–14 years: Asthma current asthma, severe asthma, and exercise-induced asthma 6–7 years: Increased EPIC diet scores were associated with a higher risk of severe asthma (odds ratios = 2.26, 95% CI: 1.21–4.22 in the 2nd quartile, but not in the 3rd and 4th) in girls. There was no significant relationship for the other asthma categories
**Diet inflammatory index**		
De Castro et al. (116)	Portugal *N* = 501 Age assessed: 7–12 years	Asthma The effect of indoor pollution on asthma outcomes was more severe in those with a pro-inflammatory diet (OR = 1.44, 95% CI: 1.01–2.21; and OR = 1.29, 95% CI: 1.03–1.68, respectively) compared to those having an anti-inflammatory diet. No direct effect of DII on asthma outcomes were reported.
Han et al. (117)	USA *N* = 8,175 Age assessed: 6–17 years	Current asthma, current wheeze, lung function measures Higher Diet inflammatory index scores were associated with high fractional exhaled nitric oxide (a marker of eosinophilic airway inflammation; OR = 2.38, 95% CI = 1.13–5.02; *P*_trend_ = 0.05) in children. The DII was not associated with lung function or current asthma

### The Diet Inflammatory Index

Two studies investigated the role of the diet inflammatory index on allergy development in the children (116, 117) and neither one of these could find an association between diet inflammatory scores and asthma in the child. One study found that indoor pollution on asthma outcomes was more severe in those with a pro-inflammatory diet than those following an anti-inflammatory diet (116).

### The Mediterranean Diet Index

Three studies found reduced allergy outcomes with an increase in mediterranean diet scores: reduced current wheeze (107), asthma ever, wheezing ever, current sneezing, and current itchy-watery eyes (108), and severe asthma in girls (109). Five studies found no effect on any of the outcomes studied (110–114) and one study found an increased risk of severe asthma, with increased mediterranean diet scores (115).

## Conclusions

Based on the current evidence, we recommend the introduction of solid foods, including common allergenic foods, during the 1st year of life, according to the infant's neuro-developmental abilities and familial or cultural habits.

In infants with severe AD and/or FA, medical assessment may be advisable before introducing common food allergens into the diet. Consuming a diverse range of foods in the 1st year of life may increase intake of nutrients and positively affect the gut microbiome composition. Intake of omega-3 fatty acids and fibers/prebiotics may be particularly important, but more information is required about dose and which individuals are most likely to benefit. Increased diet variety in the first year of life is also associated with reduced food allergy outcomes. Limited evidence exist about the role of diet indices in pregnancy and allergic disease in the offspring. The most promising results indicate a reduction in childhood wheeze and/or asthma. Further studies are warranted to investigate the effects of diet diversity during pregnancy and lactation and diet indices in early life on the development of allergic diseases in infants and children.

## Author Contributions

ED'A conceptualized the paper, wrote the introduction, and the section on diet diversity. DP critically reviewed the draft and proofread the paper. MS wrote the abstract and made tables on diet diversity. EV contributed to write the section on diet diversity. GZ made final revisions of the draft. CV wrote the section on diet indices, made tables on diet indices, and critically reviewed the paper. All authors contributed to the article and approved the submitted version.

## Conflict of Interest

The authors declare that the research was conducted in the absence of any commercial or financial relationships that could be construed as a potential conflict of interest.
